# The Clock Drawing Test: A review of its accuracy in screening for dementia

**DOI:** 10.1590/S1980-57642009DN30200002

**Published:** 2009

**Authors:** Ivan Aprahamian, José Eduardo Martinelli, Anita Liberalesso Neri, Mônica Sanches Yassuda

**Affiliations:** 1 MD, MSc, Psychogeriatric Unit, Laboratory of Neuroscience (LIM 27), Department and Institute of Psychiatry, Faculty of Medicine, University of São Paulo, SP, Brazil. Gerontology Division, University of Campinas.; 2 MD, MSc, PhD, Assistant Professor, Geriatrics Division, Jundiaí Medical School, Jundiaí, SP, Brazil.; 3 PhD, Professor, Gerontology, Medical Sciences Faculty, UNICAMP, São Paulo, SP, Brazil.; 4 PhD, Assistant Professor of Gerontology, School of Arts, Sciences and Humanities (EACH), University of São Paulo. Psychogeriatric Unit, Laboratory of Neuroscience (LIM 27), Department and Institute of Psychiatry, Faculty of Medicine, University of São Paulo, São Paulo, SP, Brazil.

**Keywords:** clock drawing test, dementia, elderly

## Abstract

The Clock Drawing Test (CDT) is a simple neuropsychometric instrument that can be easily applied to assess several cognitive functions. Over the past 20 years, the CDT has aroused considerable interest in its role for the early screening of cognitive impairment, especially in dementia. Although the CDT is considered an accurate test for dementia screening, recent studies including comparisons with structured batteries such as the CAMCOG have shown mixed results.

**Objectives:**

To investigate the importance of the CDT compared to other commonly used tests, in the diagnosis of dementia in the elderly; (2) to evaluate the reliability and correlation between available CDT scoring scales from recent studies.

**Methods:**

A systematic search in the literature was conducted in September 2008 for studies comparing CDT scoring systems and comparing the CDT with neuropsychiatric batteries.

**Results:**

Twelve studies were selected for analyses. Seven of these studies compared CDT scoring scales while five compared the CDT against the CAMCOG and the MMSE. Eight studies found good correlation and reliability between the scales and the other tests.

**Conclusion:**

Despite the mixed results in these studies, the CDT appears to be a good screening test for dementia.

## Abstract (pt)

O Teste do Desenho do Relógio (TDR) é um instrumento neuropsiquiátrico simples que pode ser aplicado facilmente para avaliar diversas funções cognitivas. Nos últimos 20 anos, o TDR tem despertado interesse considerável sobre seu papel no rastreio precoce de declínio cognitivo, especialmente na demência. Apesar do TDR ser considerado um bom teste para o rastreio da demência, estudos recentes com comparações com baterias neuropsiquiátricas como o CAMCOG mostram resultados variados.

**Objetivos:**

Investigar a importância do TDR quando comparado com outros testes comumente u tilizados no diagnóstico de demência; avaliar a confiabilidade e a correlação entre escalas de pontuação do TDR em estudos recentes.

**Métodos:**

Uma busca sistemática na literatura foi concluída em setembro de 2008, incluindo estudos que compararam sistemas de pontuação de TDR e que compararam o TDR com baterias neuropsicométricas.

**Resultados:**

Doze estudos foram selecionados para análise. Sete compararam escalas de pontuação de TDR e cinco compararam o TDR com o CAMCOG e o MEEM. Oito estudos encontraram boa correlação e confiabilidade entre as escalas e outros testes.

**Conclusão:**

Apesar dos resultados variados nesses estudos, o TDR parece ser um bom teste de rastreio cognitivo para demência.

The Clock Drawing Test (CDT) is a simple neuropsychometric instrument that can be easily applied to assess several neuropsychiatric functions.^1^ The CDT was introduced in the early 20^th^ century as an indicator of constructional apraxia.^2^ From 1953 to mid-1986, the CDT was mainly used to screen visuoconstructional disorders associated with lesions in the parietal region of the brain.^3^ Constructional apraxia may occur in many neurological diseases, such as in patients with stroke sequelae, and is often present in early dementia.^4-6^ Over the past 20 years the CDT has aroused considerable interest for its role in early screening of cognitive impairment, especially in Alzheimer’s disease.^7-19^

In 1986, Shulman et al. published the first study associating the CDT with the screening of elderly patients with cognitive disorders, particularly the screening and follow-up of acute dementia and delirium.^13^ Since then, various studies have been carried out with the aim of establishing criteria to apply and interpret the CDT and evaluate its current role as a screening instrument for patients with cognitive impairment.^1^ Its contribution has also been investigated in the assessment and follow-up of delirium, focal cerebral lesions, Huntington’s disease, schizophrenia, unilateral neglect, multiple sclerosis, among others.^1^

In broad terms, the test evaluates several cognitive skills, similarly to the Mini-Mental State Examination (MMSE).^20^ Many cortical, subcortical, anterior, posterior, right and left skills in brain hemispheres have to operate simultaneously to draw a clock, particularly involving the frontal, temporal and parietal regions.^3^ This makes the CDT an interesting instrument for identification and follow-up of patients with possible dementia.^3^ The test assesses many cognitive skills that may be involved in early Alzheimer’s disease, such as short term memory, understanding of verbal instructions, spatial orientation, abstract thinking, planning, concentration, executive and visuospatial skills.^3^

Our aim in this study was:

(1) to investigate the importance of the CDT test compared to other commonly used tests, in the diagnosis of dementia in the elderly;
(2) to evaluate the reliability of and correlation among available CDT scoring scales based on results of recent studies.

## Methods

A systematic search of the literature was conducted (in September 2008) for articles comparing CDT scoring systems and the CDT with other neuropsychiatric instruments in dementia. A search for relevant publications was carried out of the PubMed (1950–2008) and the PsycInfo (1806–2008) databases to identify studies reporting on clock drawing test and dementia. Keywords used in the systematic search were: “clock drawing”, “clock test”, “screening”, “accuracy”, “scales”, “cognitive impairment”, “mild cognitive impairment”, “dementia”, “Alzheimer’s disease”, “old age,” “elderly”. The results were limited to articles published in English and which were based on human research. The references of key articles or books were also examined for citations missed by the search strategy. Each article resulting from this search was analyzed by the authors in a search for comparisons among CDT scoring scales and between CDT scales and other neuropsychometric batteries. Articles published after the systematic review of the CDT (Shulman, 2000)^1^ were of special interest to this study.

## Results

The initial search strategy conducted in September 2008 identified 115 potentially relevant papers regarding CDT and dementia. An initial review of the abstracts excluded 79 papers because they did not compare CDT scoring scales or the CDT with other instruments. Eleven articles were selected from the remaining papers. One study (Bourke et al., 1995)^21^ was selected from the references of the previously selected papers. Seven papers involved CDT scoring comparisons (Table 1) and five studies compared the CDT with the Cambridge Cognitive Examination (CAMCOG) and the MMSE (Table 2).

**Table 1:** Studies comparing CDT scales in screening for dementia.

Study	Scales	Population	Conclusion
Storey et al. (2001)^27^	Shulman et al. (1993) Mendez et al. (1992) Sunderland et al. (1989) Wolf-Klein et al. (1989) Watson et al. (1993)	Dementia (n=72) No dementia (n=55)	Good reliability, but with lower accuracy than previously reported for the scales.
Richardson and Glass (2002)^31^	Shulman et al. (1993) Mendez et al. (1992) Sunderland et al. (1989) Wolf-Klein et al. (1989) practical scale developed by authors	AD, VD, mixed dementia (n=63)	Good correlation between the MMSE and CDT. The Shulman and practical scales performed best.
Schramm et al.(2002)^28^	Sunderland et al. (1989) Manos e Wu (1994) Wolf-Klein et al. (1989) Shulman et al. (1989) Watson et al. (1993)	Dementia (n=79) Controls (n=44)	Good correlation between CDT, MMSE and SKT. Association between CDT and MMSE or SKT improved screening.
Seigerschmidt et al.(2002)^34^	Manos e Wu (1994) Wolf-Klein et al. (1989) Shulman et al. (1989) Watson et al. (1993)	Community elderly (health and cognitive impaired) (n=253)	Poor correlation between CDT, MMSE, verbal fluency and SKT. Poor detection for dementia with CDT.
Scalan et al. (2002)^19^	Shulman et al. (1986) Mendez et al. (1992) Sunderland et al. (1989) Wolf-Klein et al. (1989) Manos e Wu (1994) Lam et al. (1998) CERAD system	AD (n=80)	Naive raters were almost as good as trained raters. Results from complex scales like Mendez were similar to simpler scales such as CERAD.
Powlishta et al. (2002)^38^	Rouleau et al. (1992) Manos e Wu (1994) Mendez et al. (1992) AD Cooperative Study (1999) Pfizer Inc. (1997) Sunderland et al. (1989)	AD (n=60) Controls (n=15)	Low sensitivity for very mild AD. All scales were similar in the detection of AD.
Connor et al. (2005)^36^	Wolf-Klein et al. (1989) Rouleau et al. (1992) Watson et al. (1993)	AD (n=50) Controls (n=50)	Good reliability between the three scales only for moderate and severe AD.

AD, Alzheimer's disease; CDT, Clock Drawing Test; MMSE, Mini-Mental State Examination.

**Table 2:** Studies comparing the CDT with a neuropsychiatric battery for dementia.

Study	Scales	Population	Conclusion
Bourke et al. (1995)^21^	Shulman et al. (1993), Mendez et al. (1992)×CAMCOG and the pentagon drawing	AD (n=77)	Good reliability but with high false-negatives.
Heinik et al. (2002)^47^	Shulman et al. (1993), Freedman et al. (1994)×CAMCOG e MMSE	AD (n=49)	Good correlation between the CDT and the CAMCOG in mild AD. Poor correlation between Freedman and MMSE and CAMCOG in CDR 2 patients
Heinik et al.(2003)^48^	Freedman et al. (1994)×CAMCOG and MMSE	Dementia (n=88) Depression and anxiety disorders (n=26)	Good correlation between the CDT, MMSE and the CAMCOGCDT plus MMSE were almost as good as the CAMCOG
Heinik et al. (2004)^16^	Shulman et al. (1993), Freedman et al. (1994), CAMCOG scale×CAMCOG and MMSE	AD (n=52)VD (n=36)Depression and anxiety disorders (n=26)	Good correlation between the CDT and the MMSE and CAMCOG.
Van der Burg et al. (2004)^49^	Shulman et al. (1993), CAMCOG scale×CAMDEX	Dementia (n=387)Controls (n=86)	Weak reliability with Shulman scale. Low specificity in both scales.

AD, Alzheimer's disease; CDT, Clock Drawing Test; MMSE, Mini-Mental State Examination.

## Discussion

### CDT scoring scales

There are more than fifteen well validated scales to interpret the CDT. They provide qualitative^12,13,22^ or quantitative^10,11,23,24^ methods of variable complexity. These scales are based on (1) strict and well-structured protocols, (2) the most frequent findings after test application, and (3) opinion of experts.^1^

There is no consensus in the literature about which scale is the most adequate for test interpretation. Shulman^1^ found mean sensitivity and specificity levels of 85% across all scales by using a statistical instrument to group all scales according to a similar scientific method. Conceptual opposition to these findings remains in the literature due to difficulties in replicating the results (Table 1).^25-29^

Comparisons among scales have been questioned, since the studies showed major methodological differences in terms of patient recruitment and clinical procedures and presence or absence of comparison with instruments of higher diagnostic accuracy.^30^

Despite such limitations, several studies in the literature have indicated that the scales by Shulman et al.,^13^ Mendez et al.^24^ and Sunderland et al.^10^ showed greater diagnostic accuracy and similar results when compared with neuropsychiatric exams, even when used in populations with diverse cultural backgrounds and educational levels.^1,18,19,30,31^

A study in Brazil by Shulman et al.^13^ sought to evaluate intra and inter–rater reliabilities of the CDT scored (scores from 0 to 5; cut-off: 3 points) by two independent raters, in an elderly random sample of 202 subjects with very low formal educational level.^15^ Intra and inter–rater reliabilities were excellent when CDTs were classified as ‘normal’ (scores 4 or 5) or ‘abnormal’(scores 0 to 3) (kappa=0.99 and 0.94, respectively) and were in the good to excellent range when scored from 0 to 5 (kappa=0.88 and 0.74, respectively).^15^

Storey et al. compared the scales of Shulman et al.,^22^ Mendez et al.,^24^ Sunderland et al.,^10^ Wolf-Klein et al.,^12^ and Watson et al.^23^ in elderly individuals with clinical diagnosis of dementia according to the DSM-IV.^32^ Inter-rater reliability was high for all five scales (0.81–0.93), although they found lower accuracy than original studies.^27^ The methods by Shulman et al.^22^ and Mendez et al.^24^ demonstrated the best diagnostic accuracy.^27^

Richardson and Glass, in a study of 63 patients with Alzheimer’s disease, vascular and mixed dementia, analyzed five CDT scales (Shulman et al.,^22^ Mendez et al.,^24^ Sunderland et al.,^10^ Wolf-Klein et al.,^12^ and a practical scale developed by one of the authors) and found robust correlation between the MMSE and the CDT in all scales.^31^ Another study also showed significant correlations among five similar methods of CDT analysis and the MMSE and Short Performance Test (SKT).^28^ Scalan et al. compared six CDT scales (Shulman et al.,^22^ Mendez et al.,^24^ Sunderland et al.,^10^ Wolf-Klein et al.,^12^ Manos and Wu,^33^ Lam et al.35) for scores obtained from naive and experienced raters, who had classified as normal or abnormal on the CDT.^19^ Surprisingly, three of the scales (Sunderland, Wolf-Klein, Lam) showed poorer performance than that of the clocks assessed by experienced raters.^19^ Finally, a study involving Alzheimer’s patients and controls used the scales by Wolf-Klein et al.,^12^ Rouleau et al.,^9^ and Watson et al.^23^ and found good inter-rater reliability,^36^ although all CDTs lacked sensitivity in milder dementia.^36^

Studies involving patients at early stages of dementia are still rare in the literature.^1^ A study involving patients with mild cognitive impairment and questionable dementia showed a weak relationship between the CDT and the MMSE, SKT and verbal fluency tests, but a strong correlation between four scales (Manos and Wu,^33^ Wolf-Klein et al.,^12^ Shulman et al.^22^ and Watson et al.23).^34^ Lee et al. conducted a study involving 30 patients at early stages of Alzheimer’s disease and 30 normal patients.^37^ The CDT was analyzed using the scales by Sunderland et al.^10^ and Mendez et al.^24^ The patients with Alzheimer’s disease were classified according to the disease stage as CDR 0.5 (very mild), 1.0 (mild) or 2.0 (moderate). The CDT sensitivity was lower for patients with CDR 0.5 on both scales (Sunderland, mean 67%; 33% for CDR 0.5, 77% for CDR 1.0 and 100% for CDR 2.0; Mendez, mean 73%; 44% for CDR 0.5, 82% for CDR 1.0 and 100% for CDR 2.0). All the clocks were compared with the CAMCOG battery to evaluate their performance.^37^ The only significant correlation was between the CAMCOG praxis rating and the Sunderland scale.^37^

In a longitudinal study, patients with initial to advanced Alzheimer’s disease were evaluated.^38^ A total of 75 patients were selected: 15 normal controls, 25 with very mild dementia (CDR=0.5), 21 with mild dementia (CDR=1.0), and 14 with moderate and severe dementia (CDR=2.0 or 3.0). Each CDT was blindly rated by two raters using six standardized scales. The same scales for the CDT interpretation were used for follow-up. All scales had low sensitivity in identifying individuals at early stages of dementia, allowing for a significant number of false positives.^38^

A recent study has analyzed the most common errors found on the CDT in a population of 536 elderly individuals, developing an interpretation scale based on the most frequent errors.^39^ This scale showed that six errors are needed for good discrimination between normal elderly individuals and those with dementia, and that the error scale may be better than the three scales most frequently used in the literature.^39^

### Comparison between the Clock Drawing Test and other instruments or batteries for cognitive screening

The CDT is a screening instrument with sensitivity and specificity approaching that of the MMSE (87 and 86%, respectively).^40^ The correlation between the CDT and the MMSE ranges from moderate (0.30) to high (0.77), mean 0.61.^1^ The highest correlations were found for the scale by Shulman et al.,^22^ Mendez et al.^24^ and the CLOX scale.^41^ Comparison of correlation between the MMSE and other cognitive screening tests range between 0.60–0.90, higher than those for the CDT.^40^

Brodaty and Moore showed that the clock test can be better than the MMSE at a memory clinic.^30^ There is also a potential advantage when both tests are applied concomitantly.^42^ The MMSE includes limited assessment of visuospatial and executive functions which may be altered in some dementia patients at early stages of the disease more prominently than language and memory.^5^

Juby conducted a study with 150 elderly outpatients at a general clinic comparing the MMSE and three interpretation methods of the CDT.^43^ The researcher used the scales by Sunderland et al.^10^, Wolf-Klein et al.^12^ and Watson et al.^23^ All CDT scores were significantly associated with the MMSE results showing high to moderate correlations (p=0.01) ranging from –0.50 to 0.67.^43^ A study including normal controls, patients with dementia or depression was compared with both tests.^29^ In case of an abnormal result in one of the tests when the CDT and the MMSE were used together, 39 out of 41 cases of dementia were identified correctly generating a sensitivity of 95%. However, 26% of patients without dementia or depression and 30% of those with depression had lower than normal scores on one of the tests, resulting in 74 and 70% specificity, respectively. The CDT had 76% sensitivity and 81% specificity,^29^ lower than the values found in previous studies in which patients were selected from clinics specialized in neurology, memory and psychiatry.^11,12,24,30^

In a large study conducted in England, 13,557 elderly individuals completed the CAMCOG CDT (scored from 0 to 4) and the MMSE.^44^ The authors showed a 76.5% sensitivity and 87.1% specificity for moderate to severe cases of cognitive impairment with a cut-off point of two points, corresponding to an MMSE score of 17 for nurse administration and 40% sensitivity and 91% specificity for postal administration.^44^ No relationship was found in cases of mild dementia.

Solomon et al. combined the CDT with episodic memory, orientation and verbal fluency tests lasting seven minutes and found 100% sensitivity and specificity in the differentiation between likely Alzheimer’s patients and healthy controls.^45^ Scanlan and Borson associated the CDT with three memory items forming the Mini-Cog test and achieved high sensitivity (97%) and specificity (95%) in the cognitive screening of dementia.^46^ Schramm et al. combined the MMSE or the SKT with five different clocks.^28^ The sensitivity of each clock was improved to levels of up to 92% using the SKT and CDT evaluated according to Shulman et al.^28^

There are few studies comparing the CDT to neuropsychometric batteries of higher diagnostic accuracy in dementia (Table 2).^1^ Bourke et al. compared the CDT to the CAMCOG in 77 patients who met the NINCDS-ADRDA criteria for probable Alzheimer’s disease.^21^ The scales used for interpreting the clock were those by Shulman et al.^22^ and Mendez et al.^24^ There were robust correlations between the scales by Shulman (r=0.70) and Mendez (r=0.67) and the CAMCOG.^21^

The study by Heinik et al. (2002) sought to compare the scales by Shulman et al.^22^ and Freedman et al.^3^ in 49 elderly individuals with mild to moderate Alzheimer’s disease according to the MMSE and the CAMCOG.^47^ Both scales had high correlations with the CAMCOG in both stages of dementia (–0.530 to –0.733 for Shulman; 0.612 to 0.723 for Freedman) and with the MMSE only in the mild stage (–0.585 for Shulman and 0.526 for Freedman). The scale by Shulman^22^ had the same performance in mild and moderate cases, while that by Freedman^3^ showed poorer performance among patients at the moderate stage.^47^

The same authors later selected 56 patients with Alzheimer’s disease, 36 with vascular dementia and 26 controls with bipolar disorder according to the DSM-IV at a geriatric outpatient clinic.^48^ The CAMCOG was applied to all patients for comparisons with the MMSE and the CDT interpreted according to Freedman et al.^3^ The CDT showed high correlations with the MMSE (0.73) and with the CAMCOG (0.80) (*p*<0.001).^48^ The relationship between the MMSE and the CAMCOG was also high (0.93, *p*<0.001).^48^

Later, Heinik et al. analyzed three scales for CDT interpretation in the same group of patients as the previous study.^16^ They added the application of two other scales for the clock: that by Shulman et al.^22^ and the CAMCOG scale. The authors found significant correlations between the three scales (CAMCOG, Shulman, Freedman) and the CAMCOG score, as well as the MMSE.^16^ The results of Heinik et al. were better than the previous study performed by Bourke et al.^21^

Also in 2004, Van der Burg et al. conducted a study involving 473 normal controls and patients with dementia selected from the community.^49^ The CAMDEX was performed in all patients, being considered the gold standard. Two clock scales were applied: Shulman et al.^22^ and the CAMCOG scale. Inter-rater reliability was evaluated and was weak for Shulman’s scale (0.47) and high for the CAMCOG CDT (0.75).^49^ When inter-rater diagnostic agreement was evaluated, the results were much better for both scales (0.88 and 0.91, respectively). Sensitivity and specificity were similar between the scales: 97 and 32%, respectively, for the CAMCOG CDT, and 96 and 42% for the scale developed by Shulman.^49^

In conclusion, studies which tested the accuracy of the CDT in dementia screening have shown that the CDT may be scored reliably with a variety of scales and that it accurately discriminates cognitively unimpaired patients from patients showing early cognitive decline. The various interpretation scales available tend to generate congruent results and CDT scores are frequently highly correlated with other screening tests such as the MMSE and the SKT. Correlations between the CDT and more comprehensive cognitive batteries such as the CAMCOG also tend to be high. Therefore, present evidence suggests the CDT may be used as a single screening test when there are time constraints, or be applied as part of larger assessment protocols.
